# Exogenous Application of Salicylic Acid and Hydrogen Peroxide Ameliorate Cadmium Stress in Milk Thistle by Enhancing Morpho-Physiological Attributes Grown at Two Different Altitudes

**DOI:** 10.3389/fpls.2021.809183

**Published:** 2022-01-27

**Authors:** Mereen Nizar, Kanval Shaukat, Noreen Zahra, Muhammad Bilal Hafeez, Ali Raza, Abdul Samad, Qasim Ali, Manzer H. Siddiqui, Hayssam M. Ali

**Affiliations:** ^1^Department of Botany, University of Balochistan, Quetta, Pakistan; ^2^Department of Botany, University of Agriculture, Faisalabad, Pakistan; ^3^Department of Agronomy, University of Agriculture, Faisalabad, Pakistan; ^4^Key Laboratory of Ministry of Education for Genetics, Breeding and Multiple Utilization of Crops, Center of Legume Crop Genetics and Systems Biology/College of Agriculture, Oil Crops Research Institute, Fujian Agriculture and Forestry University (FAFU), Fuzhou, China; ^5^Institute of Food and Agriculture Sciences, University of Florida, Gainesville, FL, United States; ^6^Department of Botany and Microbiology, College of Science, King Saud University, Riyadh, Saudi Arabia

**Keywords:** cadmium stress, oxidative damage, chlorophyll, milk thistle, high altitude

## Abstract

Cadmium (Cd^+2^) is a potential and widespread toxic environmental pollutant, mainly derived from a rapid industrial process that has inhibitory effects on growth, physiological, and biochemical attributes of various plant species, including medicinal plants such as *Silybum marianum* L. Gaertn commonly known as milk thistle. Plant signaling molecules, when applied exogenously, help to enhance/activate endogenous biosynthesis of potentially important signaling molecules and antioxidants that boost tolerance against various abiotic stresses, e.g., heavy metal stress. The present study documented the protective role of salicylic acid (SA;0.25 μM) and hydrogen peroxide (H_2_O_2_; 10 μM) priming, foliar spray, and combinational treatments in reducing Cd^+2^ toxicity (500 μM) in milk thistle grown at two diverse ecological zones of Balochistan Province of Pakistan i.e., Quetta (Qta) and Turbat (Tbt). The morpho-physiological and biochemical attributes of milk thistle were significantly affected by Cd^+2^ toxicity; however, priming and foliar spray of SA and H_2_O_2_ significantly improved the growth attributes (root/shoot length, leaf area, and root/shoot fresh and dry weight), photosynthetic pigments (Chl *a*, *b*, and carotenoids) and secondary metabolites (Anthocyanin, Soluble phenolics, and Tannins) at both altitudes by suppressing the negative impact of Cd^+2^. However, the oxidative damage parameters, i.e., MDA and H_2_O_2_, decreased astonishingly under the treatment of signaling molecules, thereby protecting membrane integrity under Cd^+2^ stress. The morphological variations were profound at the low altitude (Tbt) as compared to the high altitude (Qta). Interestingly, the physiological and biochemical attributes at both altitudes improved under SA and H_2_O_2_ treatments, thus hampered the toxic effect of Cd^+2^. These signaling compounds enhanced tolerance of plants under heavy metal stress conditions with the consideration of altitudinal, and ambient temperature variations remain to be the key concerns.

## Introduction

Milk thistle (*Silybum marianum* L. Gaertn) is a medicinal weed belonging to the Asteraceae family. It grows well at an altitude of 1,800–2,400 m a.s.l in sandy or rocky soil. The optimum temperature for germination of milk thistle seeds ranges between 28 and 29°C. The density of plants reaches up to 4.5 plant/m^2^ (Karkanis et al., 2011; Heidari et al., 2014). Medicinal plants are of very high significance because they provide the community with health care and prevention from diseases. A rapid increase in the population and pharmaceutical industry needs medicinally important plants with active ingredients to enhance and promote their cultivation and production (Valková et al., 2021; Zahra et al., 2021c). Compounds of pharmaceutical properties are derived from milk thistle fruits, i.e., achenes. Seed and dry pericarp accumulate a large flavonolignans group called silymarin, being the precursor taxifolin (Bijak, 2017).

The environmental contamination to heavy metals and other pollutants hailing from the **eighteenth** century (Andrade et al., 2014) in which cadmium (Cd^+2^) is the most toxic heavy metal for the living biota (Raza et al., 2020). Heavy metals toxicity has been widely recognized (Raza et al., 2021a,^2022^; Salehi et al., 2021; Zahra et al., 2021a). Cd^+2^ is a non-degradable trace metal contaminant, ranked seventh among the top recognized pollutants in the environment. When Cd^+2^ enters the cells of plants, it interferes and disrupts the metabolic process because of an interaction with some organic compounds within organelles of cells and the cytosol (Ali et al., 2013; Mwamba et al., 2020; Raza et al., 2020). Furthermore, it may interact with proteins and lipids that result in affecting the enzyme and fluidity of the membrane by causing oxidative damage that initiates free radical formation (Yu et al., 2018; Mwamba et al., 2020; Raza et al., 2021b,^2022^). It binds to sulfhydryl groups in proteins that also replace the important metal ions metalloproteins (Raza et al., 2021c). The mountainous areas plants, in contrast to land plants, display changes in physiognomy, anatomy, and physiology. Plant characteristics can be divided into two groups with the elevation changes. The variations of recorded data confirmed the higher antioxidant activity of plant extracts, which were derived from low to high altitude (Spitaler et al., 2008).

Salicylic acid is widely used to promote the plants’ growth and their development under varying favorable and unfavorable conditions of the ambient environment. SA has a crucial role in mediating the responses to the toxicity of heavy metals. Recently, it has been reported that SA ameliorates Cd^+2^ stress by improving germination and protecting membrane (Lu et al., 2018; Majumdar et al., 2020). Hydrogen peroxide (H_2_O_2_) is recognized as one of the main chemicals, having the properties of inducing tolerance under biotic and abiotic stress (Dikilitas et al., 2020). H_2_O_2_ as a signaling molecule also plays a key role within plants and acts as a messenger molecule involved in signaling, which triggers stress tolerance against different stresses of abiotic conditions. Growing milk thistle across the altitudinal gradient for exploring the tolerance potential under Cd^+2^ toxicity is a pragmatic approach to exploring the mechanisms for its tolerance and survival in diverse environmental regimes. Recently, variation in morpho-physiological attributes has been reported with different milk thistle ecotypes grown at a single ecological zone under different stresses (Zahra et al., 2021b,d). However, the information about Cd^+2^ tolerance of milk thistle under priming and foliar of H_2_O_2_ and SA, grown at the diverse ecological zones, is still lacking. The data on morpho-physiological attributes of milk thistle grew at varying altitudes under Cd^+2^ stress are not reported from Balochistan, Pakistan. In the present study, an effort has been made to explore the tolerance potential of milk thistle to Cd^+2^ stress under priming and foliar treatments of H_2_O_2_ and SA grown at the Quetta and Turbat area of Balochistan, Pakistan.

## Materials and Methods

### Experimental Detail

**FIGURE 1:**
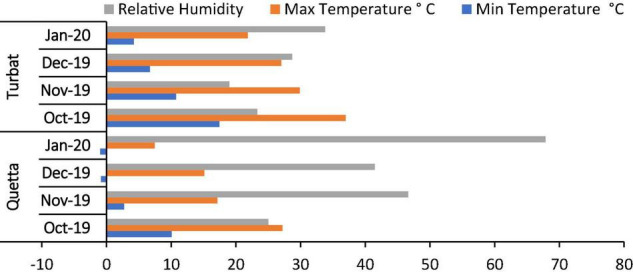
Average monthly maximum, minimum temperature, and relative humidity during the experiment period October 2019–January 2020.

A study was planned to explore/evaluate the role of H_2_O_2_ and SA in alleviating cadmium toxicity in milk thistle that was grown across the altitudinal gradient, i.e., at Quetta (1,679 m) and Turbat (129 m). Both areas have diverse environmental regimes with varying maximum and minimum temperatures around the year (Figure 1). A Randomized Complete Block Design (RCBD) experiment was designed in which the field was divided into two major plots: control and cadmium (500 μM). Each plot further contained ten treatments with three replicates. The experimental plot was 57 × 12 feet (each plot, i.e., control and cadmium) at both experimental sites. Here, control treatment means no foliar and no priming treatment in both cadmium treated and untreated plants/control plants). Prior to the sowing of seeds, the soil of both experimental sites was analyzed for its physio-chemical properties (Table 1). Seeds were sown at 1-inch depth, row to row, and plant-to-plant distance was 1 ft.

**TABLE 1:** Physio-chemical analysis of the soil of experimental sites.

Region	Exchangeable potassium	Available phosphorus	Total nitrogen	Cadmium	Organic matter	pH	Electrical conductivity

	(mg/kg)	(mg/kg)	(mg/kg)	(mg/kg)	%		(dS/m)
Quetta	241	4.9	0.275	0.36	0.502	8.05	1.52
Turbat	197	4.1	0.165	0.31	0.195	7.84	9.08
References	Bremner and Mulvaney, 1982	Olsen et al., 1954	Richards, 1954	Lindsay and Norvell, 1978	Walkley and Black, 1934	McLean, 1983	Rhoades, 1982

### Priming and Foliar Spray

Seeds of milk thistle were collected from Balochistan Agriculture Research and Development Centre (BARDC), Quetta for experiment. The seeds of milk thistle were primed in concentrations of 0.25 μM salicylic acid (SA), 10-μM hydrogen peroxide (H_2_O_2_), and with distilled water (H_2_O) for 8 h, and then seeds were sown in the field. After 15 days of germination, Cd^+2^ was applied at both altitude fields with a concentration of 500 μM. These concentrations were selected based on our preliminary trial (data not shown); thus, one suitable level was selected for the present study. In the field of cadmium, a black sheet was placed at 5-foot depth in order to avoid its leaching. After 15 days of cadmium treatment, plants were foliarly treated using the same level of SA, H_2_O_2_ treatment as used for priming (P). Moreover, after 15 days of foliar spray (FS), the milk thistle plants were harvested from both experimental sites, and data were recorded for various morpho-physiological attributes.

### Determination of Growth Attributes

Root and shoot length (cm), number of leaves, leaf area (cm^2^), root and shoot fresh (g), along with dry weight (g), were determined. Three plant samples were randomly collected for measuring root and shoot length by using a scale, and the number of total leaves/per plant was counted. Plants were harvested carefully. Before harvest, the field was fully watered to make safe and easy removal of roots from soil without any mechanical damage. The area of the leaf was calculated by following an equation developed by Cain and Castro (1959) by using the given formula, A 1/4.75 × L × W. The L described the length of the leaf, while W showed the width of the leaf, and 0.75 was being utilized for the correction factor that was used for conservation of the leaf length and width rectangular product into the area of the leaf. Lastly, three roots and shoots were separately weighted to determine the fresh weight (FW) and placed in an oven (65–75^°^C, 72 h) to obtain dry weight (DW).

### Physiological Analysis

#### Photosynthetic Pigment Analysis

Leaf samples (0.1 g) were extracted with acetone (80%). The absorbance was recorded at 663 nm, 645 nm and 480 nm against 80% acetone (Arnon, 1949; Kirk and Allen, 1965). Chlorophyll *a*, *b*, and carotenoids contents were measured using the following formulas:


$$\begin{array}{c} \mathrm{Chlorophyll}\;a\left(\mathrm{mg}/g\mathrm{fresh}\mathrm{wt}.\right)=(1.27\left(\mathrm{OD663}\right)\\ \;-2.69\left(\mathrm{OD645}\right)\times V/1000\times W \end{array}$$

$$\begin{array}{c} \mathrm{Chlorophyll}\;b\left(\mathrm{mg}/g\mathrm{fresh}\mathrm{wt}.\right)=(22.9\left(\mathrm{OD645}\right)\\ \;-4.68\left(\mathrm{OD663}\right)\times V/1000\times W \end{array}$$

$$\begin{array}{c} \mathrm{Carotenoids}\left(\mathrm{mg}/g\mathrm{fresh}\mathrm{wt}.\right)=(\mathrm{OD480}+0.114\left(\mathrm{OD663}\right)\\ \;-0.638\left(\mathrm{OD645}\right)/2500)\times 1000 \end{array}$$

#### Determination of Anthocyanins

For anthocyanins contents determination, 0.1 g of plant sample was taken and extracted in 1–2-ml acidified methanol. The samples were placed in a water bath at 50^°^C for 1 h. The absorbance was measured at 535 nm by an spectrophotometer using acidified methanol as blank (Strack and Wray, 1989).

#### Soluble Phenolic

Soluble phenolic was determined by taking 0.1 g of a plant sample by grinding in 1–2 ml of 80% acetone. In the test tube, 1 ml of distilled water was added in 100 μL of the sample, followed by the addition of 0.5 ml folin phenol reagent, and then 2.5 ml of 20% Na_2_CO_3_ (sodium carbonate) was added. The absorbance at 745 nm was measured using 80% acetone as blank (Julkunen-Tiitto, 1985).

#### Tannins

A sample (root and shoot) was taken and added 2 ml of diethyl ether and left overnight, and then decanted the solution, and 1 ml of 70% acetone was added and kept overnight. From each sample, 50 μL of the extract was taken in each test tube, and the volume was made up to 1 ml with distilled water. After dilution, 0.5 ml of folin phenol reagent was added vortexed, and then added 2.5 ml of 20% Na_2_CO_3_ solution mixed very well and kept for 40 min at room temperature. The absorbance was recorded at 725 nm using 70% acetone as a blank.

#### Hydrogen Peroxide (H_2_O_2_)

Fresh plant samples (0.1 g) were grinded in 2 ml of 0.1% (w/v) trichloroacetic acid (TCA) under pre-chilled conditions by crushing the tissues (placed on ice bath). The homogenized material was then centrifuged at 12,000 rpm for 15 min. The supernatant (0.5 ml) was mixed with 0.5 ml of a potassium phosphate buffer (pH 7.0), and 1 ml of potassium iodide solution, thoroughly mixed, and absorbance was recorded at 390 nm. Distilled water was used as a blank (Velikova et al., 2000).

#### Malondialdehyde

Malondialdehyde (MDA) was determined by the method developed by Heath and Packer (1968). A plant sample (0.1 g) of plant fresh material was grinded in 1 ml of (1% w/v) TCA and centrifuged at 12,000 rpm for 15 min. About 1 ml of supernatant was taken and mixed with 1 ml of 0.5% thiobarbituric acid in 20% TCA [0.5% in 20% (w/v) TCA] and kept in a water bath preheated at 95^°^C for 50 min. The sample extract was cooled in an ice bath. Absorbance was measured at 532 nm and 600 nm. For comparison, 1% TCA was used as a blank. MDA contents were calculated using their absorption coefficient of 155,000 nmol/mol as:


MDA[nmolml=-1(A532-A600)/155,000]106

### Statistical Analysis

The recorded data from each parameter were analyzed statistically using “STATISTIX 8.1.” The graphs and mean standard deviation were calculated using MS. EXCEL.

## Results

### Morphological Parameters

#### Root Length

**FIGURE 2:**
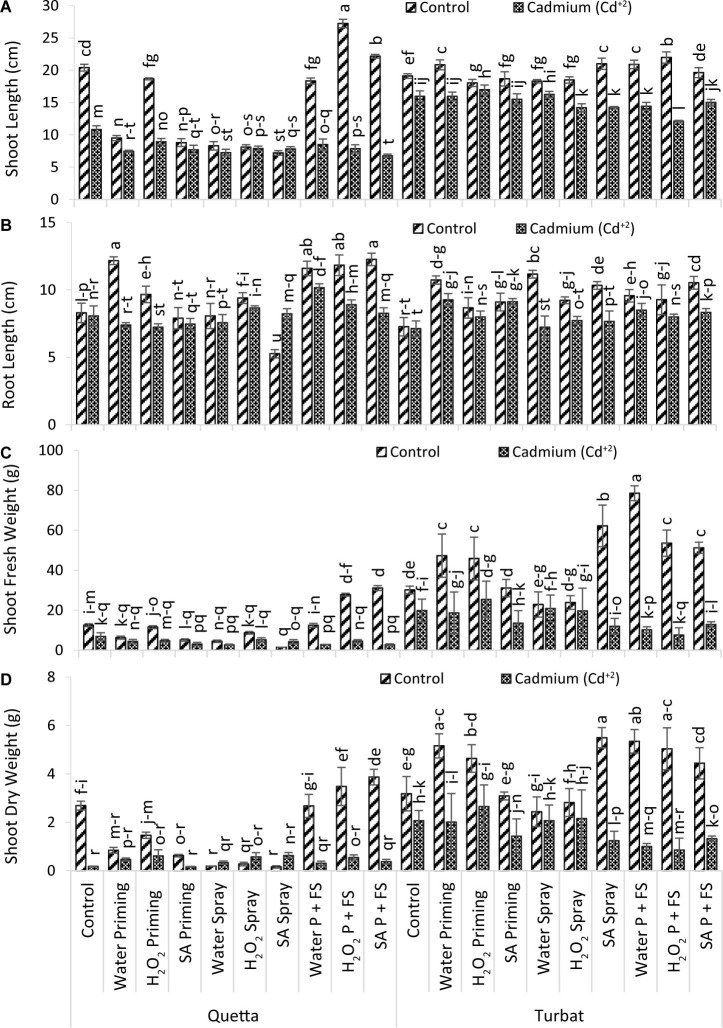
Growth parameters; shoot length **(A)**, root length **(B)**, shoot fresh weight **(C)**, shoot dry weight **(D)** of milk thistle as affected by SA and H_2_O_2_ treatment grown at Quetta and Turbat under cadmium stress. The same letters on graphs represent statistically similar effect (*p* < 0.05).

Results were obtained for the root length of milk thistle grown at two different altitudes (Qta and Tbt), under Cd^+2^, toxicity (500 μM) showed statistically significant results (Figure 2B) (*p* < 0.05). Results revealed that treatment of SA priming + foliar spray (P + FS) under control at high-altitude Qta was best, while the treatment of salicylic acid FS under control was the least effective. However, the priming and foliar spray helped to reduce the impact of Cd^+2^ toxicity in milk thistle grown at Qta and Tbt (Figure 2B).

The trend of root length at Qta was SA P + FS control > H_2_O priming control > H_2_O_2_ P + FS control > H_2_O_2_ P + FS control > H_2_O P + FS Cd^+2^ > H_2_O_2_ priming control > H_2_O_2_ spray control > H_2_O_2_ P + FS Cd^+2^ > H_2_O_2_ spray Cd^+2^ > Control of Control > SA P + FS Cd^+2^ > SA spray Cd^+2^ > Control Cd^+2^ > H_2_O spray control > SA priming control > H_2_O spray Cd^+2^ > SA priming Cd^+2^ > H_2_O P + FS Cd^+2^ > SA spray control.

Considering Tbt, the trend observed for root length was as follows: H_2_O_2_ spray control > SA P + FS control > SA spray control > H_2_O priming control > H_2_O P + FS control > H_2_O priming Cd^+2^ > H_2_O_2_ P + FS Cd^+2^ > H_2_O_2_ spray control > SA priming Cd^+2^ > SA priming control > H_2_O_2_ priming control > H_2_O P + FS Cd^+2^ > SA P + FS Cd^+2^ > H_2_O_2_P + FS Cd^+2^ > H_2_O_2_ priming Cd^+2^ > H_2_O_2_ spray Cd^+2^ > SA spray Cd^+2^ > Control of Control > H_2_O spray Cd^+2^ > control Cd^+2^ (Control; no foliar no priming treatment in both cadmium-treated and -untreated plants/control plants).

In a nutshell, the root length of milk thistle at both altitudes affected under Cd^+2^ toxicity; however, the priming and foliar application of Hydrogen peroxide (H_2_O_2_) and salicylic acid (SA) helped significantly to reduce the impacts of Cd^+2^ toxicity in milk thistle (Figure 2B).

#### Shoot Length

Data recorded for shoot length of milk thistle grown at two different altitudes (Qta and Tbt) under Cd^+2^ toxicity (500 μM) reported statistically significant results (*p* < 0.05) in which H_2_O_2_ P + FS treatment at high-altitude Qta under control conditions produced plants with greater shoot length while minimum shoot length was observed in SA P + FS of Qta under Cd^+2^ stress. Milk thistle at low altitude (Tbt) had healthy plants as compared to Qta, which demonstrated that the temperature of Tbt supported enhancing the growth of plants’ ambient conditions (Figure 2A).

The order of improvement at Qta was observed as H_2_O_2_ P + FS control > SA P + FS control > Control of Control > H_2_O_2_ priming control > H_2_O P + FS control > control Cd^+2^ > H_2_O priming control > H_2_O_2_ priming Cd^+2^ > SA priming control > H_2_O P + FS Cd^+2^ H_2_O_2_ P + FS Cd^+2^ > SA spray Cd^+2^ > SA priming Cd^+2^ > H_2_O priming Cd^+2^ > H_2_O spray Cd^+2^ > SA spray control > SA P + FS Cd^+2^.

However, the order for growth in Tbt was H_2_O_2_ P + FS control > SA spray control > H_2_O P + FS control > H_2_O priming control > SA P + FS control > Control of Control > SA priming control > H_2_O_2_ spray control > H_2_O spray control > H_2_O_2_ priming control > H_2_O_2_ priming Cd^+2^ > H_2_O spray Cd^+2^ > control Cd^+2^ > H_2_O priming Cd^+2^ > SA priming Cd^+2^ > SA P + FSCd^+2^ > H_2_O P + FS Cd^+2^ > SA spray Cd^+2^ > H_2_O_2_ Cd^+2^ > H_2_O_2_ P + FS Cd^+2^.

Overall, it has been observed that the priming and foliar spray with SA and H_2_O_2_ decreased the toxic effects of Cd^+2^ in milk thistle. However, in control conditions, both plant signaling molecules helped to significantly enhance the shoot length (Figure 2A).

#### Number of Leaves

**FIGURE 3:**
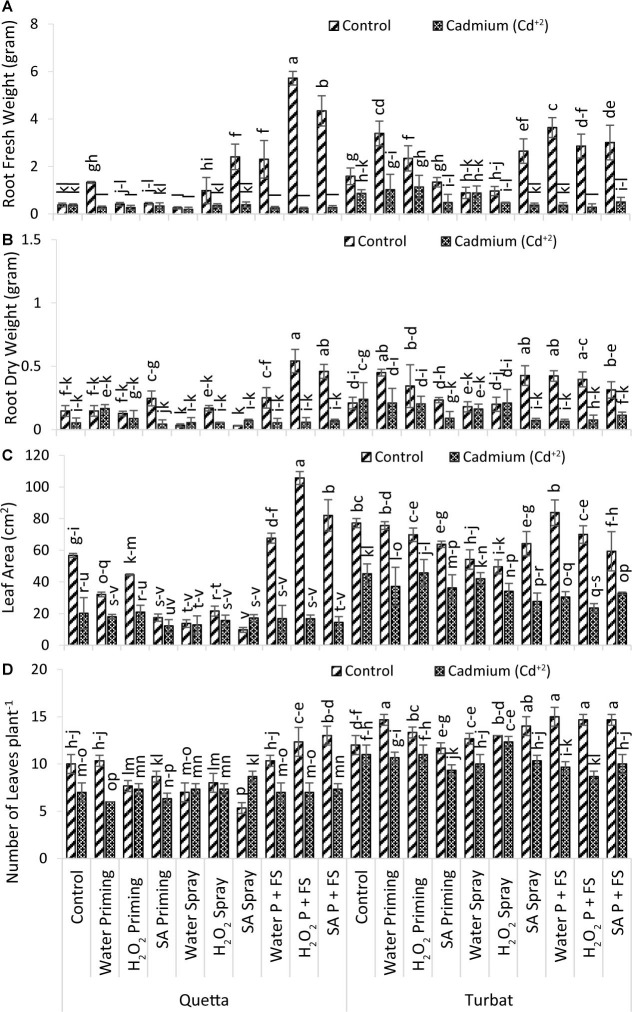
Growth parameters; root fresh weight **(A)**, root dry weight **(B)**, leaf area **(C)**, no of leaves plant^– 1^ **(D)** of milk thistle as affected by SA and H_2_O_2_ treatment grown at Quetta and Turbat under cadmium stress. The same letters on graphs represent statistically similar effect (*p* < 0.05).

Results obtained for the number of leaves of milk thistle plants grown at both the altitudes of Qta along with Tbt under the concentration of 500-μM Cd^+2^ stress displayed highly significant (*p* < 0.05) data (Figure 3D). Data for leaves count from respective treatment revealed that the highest number of leaves was recorded from H_2_O P + FS under control conditions at low-altitude Tbt, while the less number of leaves counted from SA spray under control of Qta (Figure 3D).

In Qta, the order was observed as SA P + FS control > H_2_O_2_ P + FS control > H_2_O priming control > H_2_O P + FS control > Control of Control > SA priming control > SA spray Cd^+2^ > H_2_O_2_ spray control > H_2_O_2_ priming control > H_2_O_2_ spray Cd^+2^ > SA P + FS Cd^+2^ > H_2_O_2_ priming Cd^+2^ > H_2_O spray Cd^+2^ > H_2_O_2_ P + FS Cd^+2^ > control Cd^+2^ > H_2_O spray control > H_2_O P + FS Cd^+2^ > SA priming Cd^+2^ > H_2_O priming Cd^+2^ > SA spray control.

While the order of treatments at Tbt was denoted as H_2_O P + FS control > H_2_O priming control > H_2_O_2_ P + FS control > SA P + FS control > SA spray control > H_2_O_2_ priming control > H_2_O_2_ spray control > H_2_O spray control > H_2_O_2_ spray Cd^+2^ > Control of Control > SA priming control > control Cd^+2^ > H_2_O_2_ priming Cd^+2^ > H_2_O priming Cd^+2^ > SA spray Cd^+2^ > H_2_O spray Cd^+2^ > SA P + FS Cd^+2^ > H_2_O P + FS Cd^+2^ > SA priming Cd^+2^ > H_2_O_2_ P + FS Cd^+2^.

Considering the total number of leaves among the treatments of both altitudes, it has been observed that, under control conditions, milk thistle was reported to have flourished well as compared to Cd^+2^ stress, while H_2_O_2_ and SA priming with foliar spray decreased the impact of Cd^+2^ toxicity (Figure 3D).

#### Leaf Area

Data recorded for leaf area of milk thistle grew at two different altitudes (Qta and Tbt) under 500-μM Cd^+2^ concentration reported significant (*p* < 0.05) results statistically (Figure 3C). Results for the leaf area revealed that the greater leaf area was recorded from H_2_O_2_ P + FS of control at high-altitude Qta, while the lowest was observed for SA spray of control under same-altitude Qta (Figure 3C).

The order of leaf area improvement at Qta was observed as H_2_O_2_ P + FS > SA P + FS > H_2_O P + FS > Control of Control > H_2_O_2_ priming control > H_2_O_2_ spray control > H_2_O_2_ priming Cd^+2^ > Control Cd^+2^ > H_2_O priming Cd^+2^ > SA priming control > SA spray Cd^+2^ > H_2_O P + FS Cd^+2^ > H_2_O_2_ P + FS Cd^+2^ > H_2_O_2_ spray Cd^+2^ > SA P + FS Cd^+2^ > H_2_O spray control > H_2_O spray Cd^+2^ > SA priming Cd^+2^ > SA spray control.

While order of improvement at Tbt was much better as compared to Qta that is as follows: H_2_O P + FS control > Control of Control > H_2_O priming control > H_2_O_2_ P + FS control > H_2_O_2_ priming control > SA spray control > SA priming control > SA P + FS control > H_2_O spray control > SA spray control > H_2_O_2_ priming Cd^+2^ > Control Cd^+2^ > H_2_O spray Cd^+2^ > H_2_O priming Cd^+2^ > SA priming Cd^+2^ > H_2_O_2_ spray Cd^+2^ > SA P + FS Cd^+2^ > H_2_O P + FS Cd^+2^ > SA spray Cd^+2^ > H_2_O_2_ P + FS Cd^+2^.

Considering the leaf area of both altitudinal fields, results favor a considerable enhancement of the leaf area at Tbt under control as compared to Qta plants. It is also recorded that H_2_O_2_ and SA priming + foliar spray hampered the impact of Cd^+2^ toxicity much better at low-altitudinal field Tbt as compared to Qta (Figure 3C).

#### Root Fresh Weight

Data obtained for root fresh weight of milk thistle comprising at both altitudinal fields, i.e., Qta and Tbt under Cd^+2^ stress reported significant results statistically (*p* < 0.05) described in Figure 3A. Results indicated that maximum root fresh weight was observed in H_2_O_2_ P + FS treatment under control at high-altitude Qta. However, the minimum weight was recorded from H_2_O spray under Cd^+2^ stress at the same altitude (Figure 3A).

The order of improvement of Qta was observed as H_2_O_2_ P + FS control > SA P + FS control > SA spray control > H_2_O P + FS control > H_2_O priming control > H_2_O_2_ spray control > SA priming control > H_2_O_2_ priming control > SA spray Cd^+2^ > Control of Control > Control Cd^+2^ > SA priming Cd^+2^ > H_2_O_2_ spray Cd^+2^ > H_2_O priming Cd^+2^ > SA P + FS Cd^+2^ > H_2_O_2_ priming Cd^+2^ > H_2_O_2_ spray Cd^+2^ > H_2_O P + FS Cd^+2^ > H_2_O_2_ P + FS Cd^+2^ > H_2_O spray Cd^+2^.

While, under low-altitude Tbt, the order of treatment was H_2_O P + FS control > H_2_O priming control > SA P + FS control > H_2_O_2_ P + FS control > SA spray control > H_2_O_2_ priming control > Control of Control > SA priming control > H_2_O_2_ priming Cd^+2^ > H_2_O priming Cd^+2^ > H_2_O_2_ spray control > H_2_O spray control > H_2_O_2_ spray Cd^+2^ > Control Cd^+2^ > SA P + FS Cd^+2^ > SA priming Cd^+2^ > H_2_O_2_ spray Cd^+2^ > SA spray Cd^+2^ > H_2_OP + FS Cd^+2^ > H_2_O_2_ P + FS Cd^+2^.

Concludingly, data revealed that, under Qta control, there was the maximum fresh weight of the root in contrast with Tbt control. However, considering Cd^+2^, the root fresh weight of Tbt resulted in having much improvement as compared to Cd^+2^ at Qta (Figure 3A). Overall results indicate a minimum root fresh weight, among which H_2_O_2_ and SA were quite effective in enhancing the root fresh weight of milk thistle to a very significant extent at both the altitudes (Figure 3A).

#### Shoot Fresh Weight

Data recorded for shoot fresh weight of milk thistle grown at two different altitudes (Qta and Tbt) statistically reported significant (*p* < 0.05) results (Figure 2C). Results revealed that H_2_O P + FS under control of area Tbt showed greater shoot fresh weight, while the lower shoot fresh weight was observed in SA spray under control at Qta (Figure 2C).

The order of shoot fresh weight at Qta was recorded as SA P + FS control > H_2_O_2_ P + FS control > Control of Control > H_2_O P + FS control > H_2_O_2_ priming control > H_2_O_2_ spray control > Control Cd^+2^ > H_2_O priming control > H_2_O_2_ spray Cd^+2^ > SA priming control > H_2_O_2_ priming Cd^+2^ > H_2_O_2_ P + FS Cd^+2^ > H_2_O priming Cd^+2^ > H_2_O spray control > SA spray Cd^+2^ > SA priming Cd^+2^ > H_2_O P + FS Cd^+2^ > SA P + FS Cd^+2^ > H_2_O spray Cd^+2^ > SA spray control.

While, at low-altitude Tbt, the order of improvement reported was H_2_O P + FS control > SA spray control > H_2_O_2_ P + FS control > SA P + FS control > H_2_O priming control > H_2_O_2_ priming control > SA priming control > Control of Control > H_2_O_2_ priming Cd^+2^ > H_2_O_2_ spray control > H_2_O spray control > H_2_O spray Cd^+2^ > Control Cd^+2^ > H_2_O_2_ spray Cd^+2^ > H_2_O priming Cd^+2^ > SA priming Cd^+2^ > SA P + FS Cd^+2^ > SA spray Cd^+2^ > H_2_O P + FS Cd^+2^ > H_2_O_2_ P + FS Cd^+2^.

In a nutshell, the observed data for shoot fresh weight revealed that the low-altitudinal field Tbt reported highly significant variations under control and Cd^+2^ toxicity in contrast with Qta. On the other hand, the application of SA along with H_2_O_2_ helped to suppress Cd^+2^ stress and enhance shoot fresh weight of milk thistle grown at Tbt, which describes a very healthy and effective growth of milk thistle at low altitude (Figure 2C).

#### Root Dry Weight

Results obtained for root dry weight of milk thistle grown at two varying altitudes (Qta and Tbt) under Cd^+2^ toxicity reported statistically significant results (*p* < 0.05) by revealing that the highest value for root dry weight was observed in H_2_O_2_ P + FS under control at Qta, while the minimum root dry weight was obtained from SA spray in control at Qta (Figure 3B).

At Qta, the order of treatment was H_2_O_2_ P + FS control > SA P + FS control > H_2_O P + FS control > SA priming control > H_2_O_2_ spray control > H_2_O priming Cd^+2^ > H_2_O priming control > Control of Control > H_2_O_2_ priming control > H_2_O_2_ priming Cd^+2^ > SA spray Cd^+2^ > SA P + FS Cd^+2^ > H_2_O_2_ P + FS Cd^+2^ > H_2_O spray Cd^+2^ > H_2_O_2_ spray Cd^+2^ > H_2_O P + FS Cd^+2^ > Control Cd^+2^ > SA priming Cd^+2^ > H_2_O spray control > SA spray control.

While, at Tbt, the trend observed for root dry weight was as follows: H_2_O priming control > SA spray control > H_2_O P + FS control > H_2_O_2_ P + FS control > H_2_O_2_ priming control > SA P + FS control > Control Cd^+2^ > SA priming control > H_2_O priming Cd^+2^ > H_2_O_2_ spray Cd^+2^ > Control of Control > H_2_O_2_ priming Cd^+2^ > H_2_O_2_ spray control > H_2_O spray Cd^+2^ > SA P + FS Cd^+2^ > SA priming Cd^+2^ > H_2_O_2_ P + FS Cd^+2^ > SA spray Cd^+2^ > H_2_O P + FS Cd^+2^.

Concludingly, the statistical analysis for root dry weight revealed that milk thistle showed a significant variation in root dry weight at both altitudes. However, under control, SA and H_2_O_2_ proved to be effective, moreover it also reduced the impacts of Cd^+2^ toxicity (Figure 3B).

#### Shoot Dry Weight

Data obtained for shoot dry weight of milk thistle grown at two different altitudes, i.e., Qta and Tbt, revealed statistically significant (*p* < 0.05) differences under Cd^+2^ toxicity (Figure 2D). Data revealed that, under Cd^+2^ stress, SA and H_2_O_2_ supplementations were effective in increasing shoot dry weights. Maximum weight was recorded in SA spray under control at Tbt, while the least was observed in SA spray under control of Qta, which illustrates that the supplementation with signaling molecules at Tbt performed better (Figure 2D).

However, the order of improvement recorded at Qta was SA P + FS control > H_2_O_2_ P + FS control > Control of Control > H_2_O P + FS control > H_2_O_2_ priming control > H_2_O priming control > SA spray Cd^+2^ > SA priming Cd^+2^ > H_2_O_2_ priming Cd^+2^ > H_2_O_2_ spray Cd^+2^ > H_2_O_2_ P + FS Cd^+2^ > H_2_O priming Cd^+2^ > SA P + FS Cd^+2^ > H_2_O spray Cd^+2^ > H_2_O P + FS Cd^+2^ > H_2_O_2_ spray Cd^+2^ > H_2_O spray Cd^+2^ > Control Cd^+2^ > SA priming Cd^+2^ > SA spray Cd^+2^.

While, at low-altitude Tbt, the order was SA spray control > H_2_O P + FS control > H_2_O priming control > H_2_O_2_ P + FS control > H_2_O_2_ priming control > SA P + FS control > Control of Control > SA priming control > H_2_O_2_ Spray control > H_2_O_2_ priming Cd^+2^ > H_2_O spray control > H_2_O_2_ spray Cd^+2^ > Control Cd^+2^ > H_2_O spray Cd^+2^ > H_2_O priming Cd^+2^ > SA priming Cd^+2^ > SA P + FS Cd^+2^ > SA spray Cd^+2^ > H_2_OP + FS Cd^+2^ > H_2_O_2_ P + FS Cd^+2^.

Considering the overall improvement of milk thistle shoot dry weight, it can be observed that respective treatments at Tbt showed highly significant results reducing toxicity of Cd^+2^. While, at Qta, the SDW reduced to a greater extent both under control and Cd^+2^ conditions. The fluctuating temperature may have a profound effect on the growth of the milk thistle; therefore, a varying trend can be recorded in shoot dry weight (Figure 2D).

### Photosynthetic Pigments

#### Chlorophyll *a*

**FIGURE 4:**
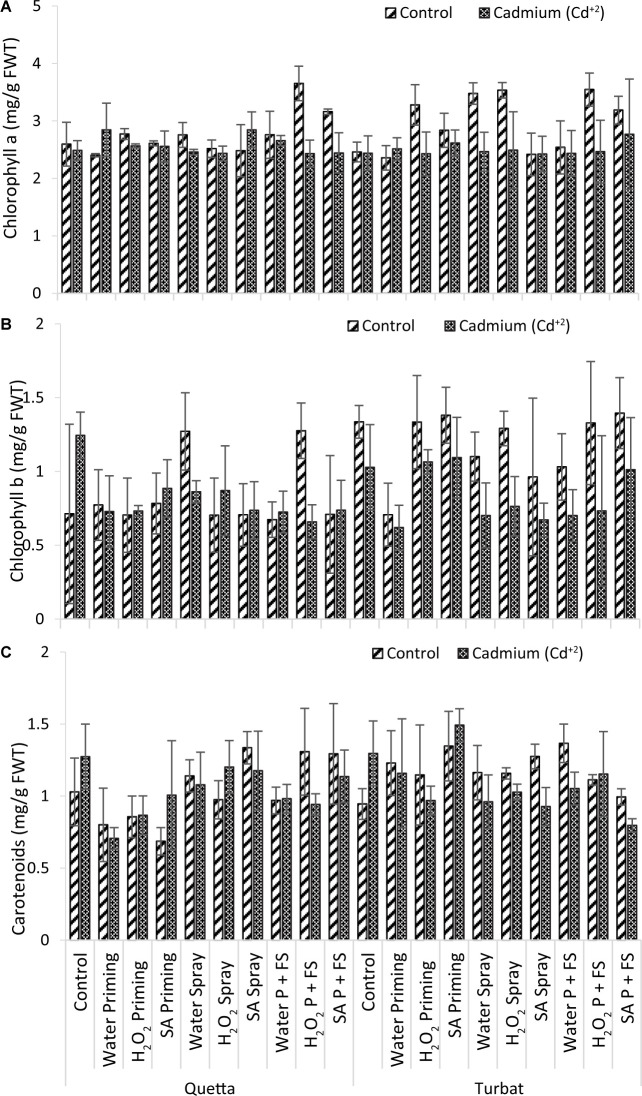
Photosynthetic pigments; Chlorophyll *a* **(A)**, Chlorophyll *b* **(B)**, carotenoids **(C)** of milk thistle as affected by SA and H_2_O_2_ treatment grown at Quetta and Turbat under cadmium stress.

Results obtained for chlorophyll *a* of milk thistle grown at two varying altitudes (Qta and Tbt) under Cd^+2^ stress of 500-μM concentration revealed statistically non-significant results (*p* > 0.05) (Figure 4A). The data further revealed that the maximum Chl *a* content was observed in H_2_O_2_ P + FS under control at Qta, while the minimum Chl *a* content was recorded in the control field at Tbt (Figure 4A).

However, the trend observed in Qta was H_2_O_2_ P + FS control > SA P + FS control > SA spray Cd^+2^ > H_2_O priming Cd^+2^ > H_2_O_2_ priming control > H_2_O P + FS control > H_2_O spray Cd^+2^ > SA priming control > H_2_O_2_ priming Cd^+2^ > SA priming Cd^+2^ > H_2_O_2_ spray control > SA spray control > Control of Control > SA P + FS Cd^+2^ > H_2_O spray Cd^+2^ > H_2_O_2_ P + FS Cd^+2^ > H_2_O_2_ spray Cd^+2^ > Control Cd^+2^ > H_2_O priming control.

While the order of improvement at Tbt was observed as H_2_O_2_ spray control > H_2_O spray control > H_2_O_2_ P + FS control > H_2_O_2_ priming control > SA P + FS control > SA priming control > SA P + FS Cd^+2^ > SA priming Cd^+2^ > H_2_O priming Cd^+2^ > H_2_O P + FS control > H_2_O spray Cd^+2^ > H_2_O_2_ spray Cd^+2^ > H_2_O_2_ P + FS Cd^+2^ > H_2_O P + FS Cd^+2^ > control Cd^+2^ > SA spray Cd^+2^ > SA spray control > H_2_O_2_ priming Cd^+2^ > H_2_O_2_ priming Cd^+2^ > H_2_O priming control > Control of Control.

Statistical data revealed a slight increase in chlorophyll *a* at both altitudes due to the priming and foliar spray of plant-signaling molecules, i.e., H_2_O_2_ and SA becoming highly helpful in reducing Cd^+2^ toxicity, thereby proving the role of signaling molecules in enhancing the content of photosynthetic pigments (Figure 4A).

#### Chlorophyll *b*

Data recorded for chlorophyll *b* of milk thistle grown at two different altitudes, i.e., Qta and Tbt, under toxicity of heavy metal (Cd^+2^) of 500-μM concentration showed statistically non-significant (*p* > 0.05) results (Figure 4B), in which data depict that SA P + FS under control of Qta was the best treatment, while H_2_O priming under Cd^+2^ stress at low-altitude Tbt proved to be the least non-significant treatment for chlorophyll *b* content (Figure 4B).

At high altitude (Qta), the trend recorded for Chl *b* was H_2_O_2_ P + FS control > H_2_O spray control > Control Cd^+2^ > SA priming Cd^+2^ > H_2_O_2_ spray Cd^+2^ > H_2_O spray Cd^+2^ > SA priming control > H_2_O priming control > SA P + FS Cd^+2^ > SA spray Cd^+2^ > H_2_O_2_ priming Cd^+2^ > H_2_O priming Cd^+2^ > H_2_O P + FS Cd^+2^ > Control of Control > SA P + FS control > SA spray control > H_2_O_2_ priming control > H_2_O_2_ spray control > H_2_O P + FS control > H_2_O_2_ P + FS Cd^+2^.

However, at low altitude (Tbt), the trend was observed as SA P + FS control > SA priming control > Control of Control > H_2_O_2_ priming control > H_2_O_2_ P + FS control > H_2_O_2_ spray control > H_2_O spray control > SA priming Cd^+2^ > H_2_O_2_ priming Cd^+2^ > H_2_O P + FS control > Control Cd^+2^ > SA P + FS Cd^+2^ > SA spray control > H_2_O_2_ spray Cd^+2^ > H_2_O_2_ P + FS Cd^+2^ > H_2_O priming Cd^+2^ > H_2_O spray Cd^+2^ > H_2_O P + FS Cd^+2^ > SA spray Cd^+2^ > H_2_O priming Cd^+2^.

Overall data statistically revealed that values for chlorophyll *b* were not significant (*p* > 0.05); therefore, it was established that, under control, the respective treatments at both the altitude (Qta and Tbt) were at their best, but the priming and foliar application of SA and H_2_O_2_ enhanced chlorophyll *b* content in milk thistle (Figure 4B).

#### Carotenoids

Results obtained for carotenoids of milk thistle, which was grown at two different altitudes (Qta and Tbt) under heavy metal stress of Cd^+2^, reported statistically non-significant results (*p* > 0.05). Data further revealed that the best treatment was SA priming under Cd^+2^ stress at Tbt, while the least carotenoids contents were observed in the SA priming treatment of control of Qta (Figure 4C).

The order of improvement at Qta was observed as SA spray control > H_2_O_2_ P + FS control > SA P + FS control > Control Cd^+2^ > H_2_O_2_ spray Cd^+2^ > SA spray Cd^+2^ > H_2_O spray control > SA P + FS Cd^+2^ > H_2_O spray Cd^+2^ > Control of Control > SA priming Cd^+2^ > H_2_O P + FS Cd^+2^ > H_2_O_2_ spray control > H_2_O P + FS control > H_2_O_2_ P + FS Cd^+2^ > H_2_O_2_ priming Cd^+2^ > H_2_O_2_ priming control > H_2_O priming control > SA priming control.

While the order at Tbt was recorded as follows: SA priming Cd^+2^ > H_2_O P + FS control > SA priming control > Control Cd^+2^ > SA spray control > H_2_O priming control > H_2_O spray control > H_2_O priming Cd^+2^ > H_2_O_2_ spray control > H_2_O_2_ P + FS Cd^+2^ > H_2_O_2_ priming control > H_2_O_2_ P + FS control > H_2_O P + FS Cd^+2^ > H_2_O_2_ spray Cd^+2^ > SA P + FS control > H_2_O_2_ priming Cd^+2^ > H_2_O spray Cd^+2^ > Control of Control > SA spray Cd^+2^ > SA P + FS Cd^+2^.

Overall data for carotenoids contents revealed that the application of SA both under control and Cd^+2^ conditions of priming and foliar spray highly improved the carotenoids contents at both varying altitudes, i.e., Qta and Tbt. However, under heavy metal concentration (Cd^+2^), the treatments showed a remarkable increase in carotenoids contents, which proves it plays a defensive role in alleviating Cd^+2^ toxicity in milk thistle (Figure 4C).

In a nutshell, considering results, it is proved that the presence of SA and H_2_O_2_ in leaves of milk thistle is highly helpful for accumulation and reduction of the toxic effects of Cd^+2^ (Figures 4A–C).

### Oxidative Damage/Stress Measurement

#### Hydrogen Peroxide (H_2_O_2_) Content

**FIGURE 5:**
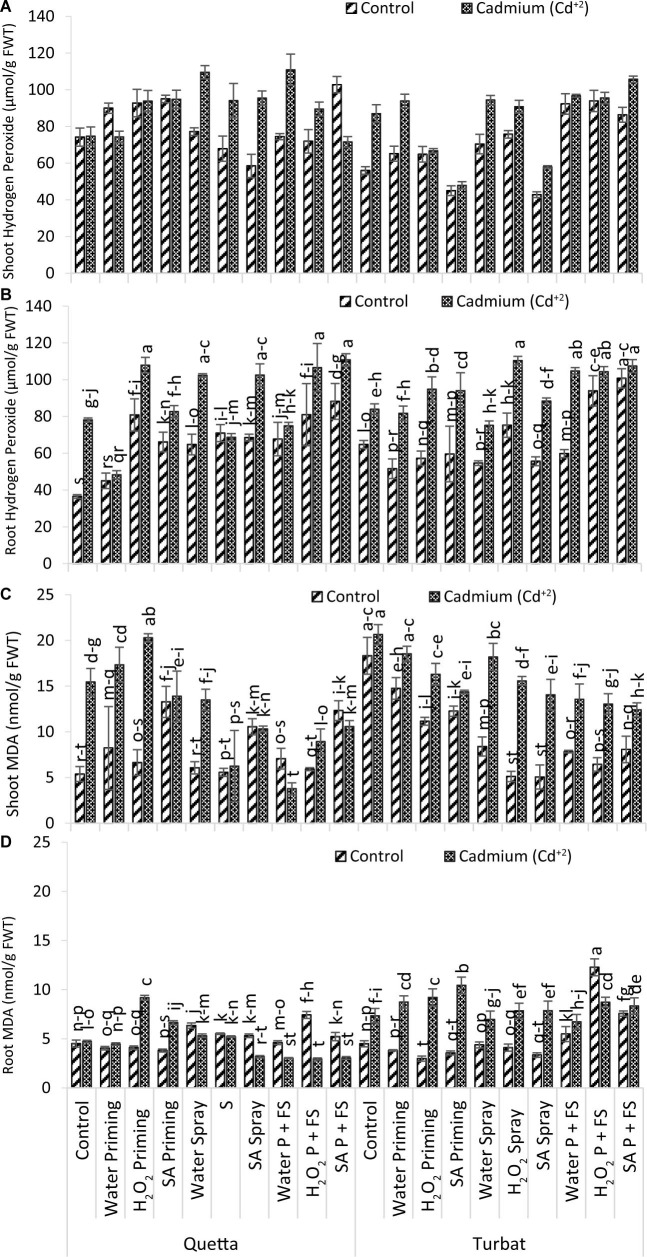
Oxidative damage parameters; shoot H_2_O_2_ **(A)**, root H_2_O_2_ **(B)**, shoot MDA **(C)**, root MDA **(D)** of milk thistle as affected by SA and H_2_O_2_ treatment grown at Quetta and Turbat under cadmium stress. The same letters on graphs represent statistically similar effects (*p* < 0.05).

Data recorded for H_2_O_2_ of milk thistle both (roots and shoots) under a concentration of 500-μM Cd^+2^ toxicity grown at two different altitudes (Qta and Tbt) in which the root showed statistically significant (p < 0.05) and shoot non-significant (p > 0.05) results (Figures 5A,B). Data for root H_2_O_2_ content revealed that highest concentration was observed in Qta and SA P + FS treatment under Cd^ + 2^ toxicity while the lowest content of H_2_O_2_ in root was observed in Control of Control of the same altitude.

The order of improvement in hydrogen peroxide (H_2_O_2_) content of root at high-altitude Qta was recorded as follows: SA P + FS Cd^+2^ > H_2_O_2_ priming Cd^+2^ > H_2_O_2_ P + FS Cd^+2^ > SA spray Cd^+2^ > H_2_O_2_ spray Cd^+2^ > SA P + FS control > SA priming Cd^+2^ > H_2_O_2_ P + FS control > H_2_O_2_ priming control > Control Cd^+2^ > H_2_O P + FS Cd^+2^ > H_2_O_2_ spray control > H_2_O_2_ spray Cd^+2^ > SA spray control > H_2_O P + FS control > SA priming control > H_2_O spray control > H_2_O priming Cd^+2^ > H_2_O priming control > Control of Control.

While, at low-altitude Tbt, the order of changes reported to be in the following sequence: H_2_O_2_ spray Cd^+2^ > SA P + FS Cd^+2^ > H_2_O P + FS Cd^+2^ > H_2_O_2_ P + FS Cd^+2^ > SA P + FS control > H_2_O_2_ priming Cd^+2^ > SA priming Cd^+2^ > H_2_O_2_ P + FS control > SA spray Cd^+2^ > Control of Cd^+2^ > H_2_O priming Cd^+2^ > H_2_O_2_ spray control > H_2_O spray Cd^+2^ > Control of Control > H_2_O P + FS control > SA priming control > H_2_O_2_ priming control > SA spray control > H_2_O spray control > H_2_O priming control.

Furthermore, results obtained for shoot H_2_O_2_ content revealed that maximum hydrogen peroxide content was observed in H_2_O P + FS under Cd^+2^ stress at Qta, while minimum content of H_2_O_2_ was observed in SA priming under control at Tbt (Figure 5A).

The order of shoot H_2_O_2_ content at Qta was recorded as H_2_O P + FS Cd^+2^ > H_2_O spray Cd^+2^ > H_2_O_2_ priming Cd^+2^ > SA spray Cd^+2^ > SA P + FS control > SA priming Cd^+2^ > H_2_O_2_ spray Cd^+2^ > H_2_O_2_ priming control > SA priming control > H_2_O priming control > H_2_O_2_ P + FS Cd^+2^ > Control of Control > H_2_O_2_ P + FS control > H_2_O priming Cd^+2^ > H_2_O P + FS control > Control Cd^+2^ > H_2_O spray control > SA P + FS Cd^+2^ > H_2_O_2_ spray control > SA spray control.

In contrast, the order of improvement at the low-altitude Tbt was recorded as follows: H_2_O_2_ P + FS Cd^+2^ > H_2_O P + FS Cd^+2^ > H_2_O P + FS control > H_2_O spray Cd^+2^ > H_2_O_2_ P + FS control > Control Cd^+2^ > SA P + FS control > H_2_O priming Cd^+2^ > H_2_O_2_ spray Cd^+2^ > H_2_O spray control > SA spray Cd^+2^ > SA P + FS Cd^+2^ > H_2_O_2_ spray control > H_2_O priming control > SA priming Cd^+2^ > H_2_O_2_ priming control > H_2_O_2_ priming Cd^+2^ > Control of Control > SA spray control > SA priming control.

Considering H_2_O_2_ content in the root of milk thistle, it was observed that the priming and foliar spray of signaling molecules, i.e., H_2_O_2_ and SA, were most effective in lowering hydrogen peroxide content in roots under Cd^+2^ toxicity. Similar findings were observed for shoots as well, proving the beneficial role of exogenous supplementation of SA and H_2_O_2_ in alleviating heavy metal toxicity (Cd^+2^). However, the interactions and trends toward hampering oxidative damage by SA and H_2_O_2_ in alleviating Cd^+2^ toxicity vary in roots and shoots (Figures 5A,B).

#### Malondialdehyde

Results obtained for MDA content of roots and shoots of milk thistle under heavy metal toxicity Cd^+2^ (500 μM) at two different altitudes, i.e., Qta and Tbt showed a significant (*p* < 0.05) difference (Figures 5C,D). Data for root MDA content revealed that the maximum MDA content was observed in H_2_O_2_ P + FS under control at Tbt, while the least was observed at Qta under Cd^+2^ toxicity, i.e., H_2_O_2_ P + FS.

However, the trend in root MDA content at Qta was observed as H_2_O_2_ priming Cd^+2^ > H_2_O_2_ P + FS control > SA priming Cd^+2^ > H_2_O spray control > H_2_O_2_ spray control > SA spray control > H_2_O spray Cd^+2^ > SA P + FS control > H_2_O_2_ spray Cd^+2^ > Control Cd^+2^ > H_2_O P + FS control > Control of Control > H_2_O priming Cd^+2^ > H_2_O_2_ priming control > H_2_O priming control > SA priming control > SA spray Cd^+2^ > H_2_O P + FS Cd^+2^ > H_2_O_2_ P + FS Cd^+2^.

While, at low altitude (Tbt), the order was recorded as H_2_O_2_ P + FS control > SA priming Cd^+2^ > H_2_O_2_ P + FS Cd^+2^ > H_2_O priming Cd^+2^ > H_2_O_2_ P + FS Cd^+2^ > SA P + FS Cd^+2^ > SA spray Cd^+2^ > H_2_O_2_ spray Cd^+2^ > SA P + FS control > Control Cd^+2^ > H_2_O spray Cd^+2^ > H_2_O P + FS Cd^+2^ > H_2_O P + FS control > Control of Control > H_2_O spray control > H_2_O_2_ spray control > H_2_O priming control > SA priming control > SA spray control > H_2_O_2_ priming control.

Furthermore, data for MDA content in the shoot of milk thistle also revealed significant data (*p* < 0.05) in which the maximum MDA content was reported in control under Cd^+2^ stress at Tbt; however, the H_2_O P + FS under Cd^+2^ stress at Qta was reported to have a minimum content of MDA (Figure 5C).

The order of changes in MDA content in the shoot at Qta was observed as H_2_O_2_ priming Cd^+2^ > H_2_O priming Cd^+2^ > Control Cd^+2^ > SA priming Cd^+2^ > H_2_O spray Cd^+2^ > SA priming control > SA P + FS control > SA P + FS Cd^+2^ > SA spray Cd^+2^ > H_2_O_2_ P + FS Cd^+2^ > H_2_O priming control > H_2_O P + FS control > H_2_O_2_ priming control > H_2_O_2_ spray Cd^+2^ > H_2_O spray control > H_2_O_2_ P + FS control > H_2_O_2_ spray control > Control of Control > H_2_O P + FS Cd^+2^.

While the trend at Tbt was recorded as follows: Control Cd^+2^ > H_2_O priming Cd^+2^ > Control of Control > H_2_O spray Cd^+2^ > H_2_O_2_ priming Cd^+2^ > H_2_O_2_ spray Cd^+2^ > H_2_O priming control > SA priming Cd^+2^ > SA spray Cd^+2^ > H_2_O P + FS Cd^+2^ > H_2_O_2_ P + FS Cd^+2^ > SA P + FS Cd^+2^ > SA priming control > H_2_O_2_ priming control > H_2_O spray control > SA P + FS control > H_2_O P + FS control > H_2_O_2_ P + FS control > H_2_O_2_ spray control > SA spray control.

Overall data of MDA content in milk thistle revealed that maximum MDA content in milk thistle had been reported in shoots both in control and Cd^+2^ conditions; in contrast, MDA content in root was reported very low. In root, the interactions of SA and H_2_O_2_ with Cd^+2^ were reported to be different. There is a significant difference in both altitudes along with the stress, i.e., Control and Cd^+2^. Considering the Cd^+2^ toxicity, it is considered that the plant-signaling molecules (i.e., SA and H_2_O_2_) reported a synergistic effect of these molecules in relation to accumulation and uptake of Cd^+2^ toxicity in the roots and shoots, thus enhancing the role of signaling molecules and suppressing the heavy metal toxicity (Figures 5C,D).

### Secondary Metabolites

#### Anthocyanin Content

**FIGURE 6:**
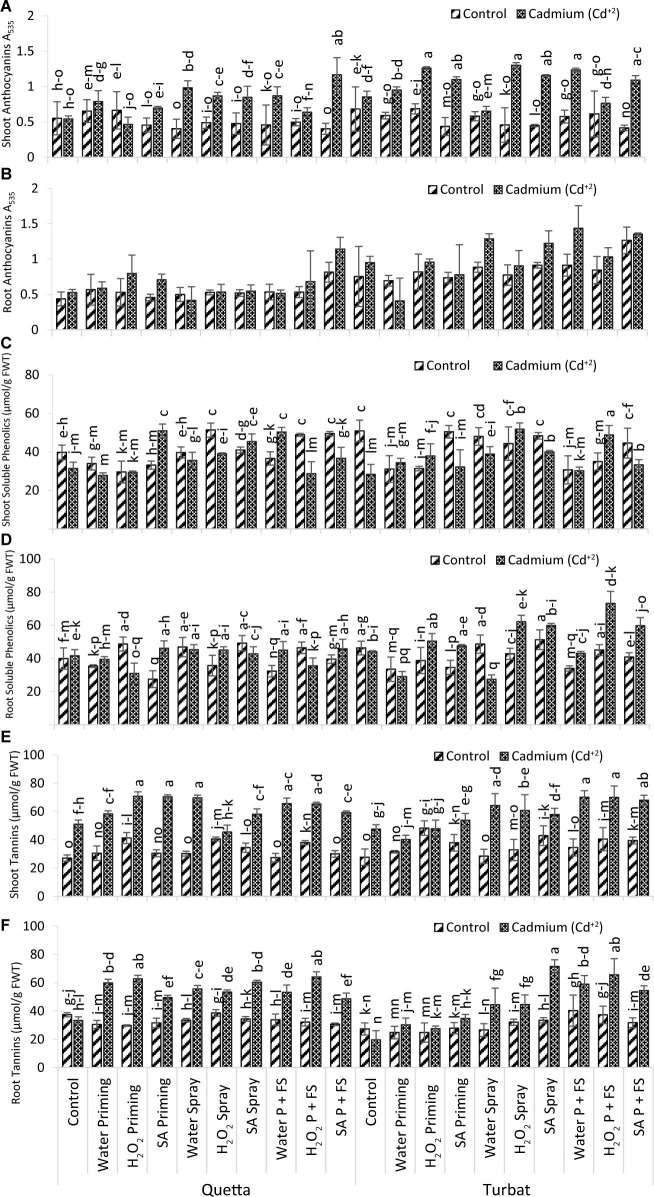
Secondary metabolites; shoot anthocyanin **(A)**, root anthocyanin **(B)**, shoot soluble phenolics **(C)**, root soluble phenolics **(D)** shoot tannins **(E)** root tannins **(F)** of milk thistle as affected by SA and H_2_O_2_ treatment grown at Quetta and Turbat under cadmium stress. The same letters on graphs represent a statistically similar effect (*p* < 0.05).

Results obtained for anthocyanin content (roots and shoots) manifested significant differences in milk thistle grown at two different altitudinal fields, i.e., Qta and Tbt under heavy metal toxicity (500 μM). Data further revealed that, in the roots, the maximum anthocyanin content was determined in H_2_O P + FS under Cd^+2^ stress at low-altitude Tbt, while the minimum anthocyanins in roots were observed in H_2_O priming under Cd^+2^ toxicity at the same altitude, showing statistically non-significant (*p* > 0.05) results (Figure 6B).

However, the trend for root anthocyanin at Qta was observed as SA P + FS Cd^+2^ > SA P + FS control > H_2_O_2_ priming Cd^+2^ > SA priming Cd^+2^ > H_2_O_2_ P + FS Cd^+2^ > H_2_O priming Cd^+2^ > H_2_O priming control > SA spray Cd^+2^ > H_2_O_2_ spray Cd^+2^ > H_2_O P + FS control > H_2_O_2_ P + FS control > H_2_O_2_ priming control > H_2_O spray control > control Cd^+2^ > SA spray control > H_2_O P + FS Cd^+2^ > H_2_O spray control > SA priming control > Control of Control > H_2_O spray Cd^+2^.

While the order of improvement at Tbt was H_2_O P + FS Cd^+2^ > SA P + FS Cd^+2^ > H_2_O spray Cd^+2^ > SA P + FS control > SA spray Cd^+2^ > H_2_O_2_ P + FS Cd^+2^ > H_2_O_2_ priming Cd^+2^ > Control Cd^+2^ > H_2_O P + FS control > H_2_O_2_ spray Cd^+2^ > H_2_O spray Cd^+2^ > H_2_O_2_ P + FS control > H_2_O_2_ priming control > SA priming Cd^+2^ > H_2_O_2_ spray control > Control of Control > SA spray control > SA priming control > H_2_O priming control > H_2_O priming Cd^+2^.

Moreover, anthocyanin content in the shoot was the highest in H_2_O_2_ spray under heavy metal toxicity (Cd^+2^) at Tbt; in contrast, SA P + FS under control remained the lowest treatment observed at Qta. The results for shoot anthocyanin content statistically showed significant (*p* < 0.05) data in milk thistle (Figure 6A).

On the other hand, the order of changes in attribute of anthocyanin contents in the shoot at Qta was recorded as SA P + FS Cd^+2^ > H_2_O spray Cd^+2^ > H_2_O P + FS Cd^+2^ > H_2_O_2_ spray Cd^+2^ > SA spray Cd^+2^ > H_2_O priming Cd^+2^ > SA priming Cd^+2^ > H_2_O_2_ priming control > H_2_O priming control > H_2_O_2_ P + FS Cd^+2^ > Control of Control > Control Cd^+2^ > H_2_O_2_ P + FS control > H_2_O_2_ spray control > SA spray control > H_2_O_2_ priming Cd^+2^ > H_2_O P + FS control > SA priming control > H_2_O_2_ spray control > SA P + FS control.

At Tbt, the order of improvement was observed as follows: H_2_O_2_ spray Cd^+2^ > H_2_O_2_ priming Cd^+2^ > H_2_O P + FS Cd^+2^ > SA spray Cd^+2^ > SA priming Cd^+2^ > SA P + FS Cd^+2^ > H_2_O priming Cd^+2^ > Control Cd^+2^ > H_2_O_2_ P + FS Cd^+2^ > H_2_O_2_ priming control > Control of Control > H_2_O spray Cd^+2^ > H_2_O_2_ P + FS control > H_2_O priming control > H_2_O spray control > H_2_O P + FS control > H_2_O_2_ spray control > SA spray control > SA priming control > SA P + FS control.

Considering anthocyanin (i.e., roots and shoots) content at two different altitudes (i.e., Qta and Tbt), it was established that there was a significant difference with the prescribed altitudes. Overall results indicate that anthocyanin content in both roots and shoots showed a small degree of differentiation statistically at Qta and Tbt under heavy metal toxicity (Cd^+2^). Thus, it proved that, along with altitudinal variations, the application of SA and H_2_O_2_ proved to be the best by reducing the toxic effect and stress of Cd^+2^. As compared to roots, there was a maximum increase in shoot anthocyanin content (Figures 6A,B).

#### Soluble Phenolics Content

Statistical analysis for roots and shoots of soluble phenolics in milk thistle grown at high (Qta) and low (Tbt) altitudes showed statistically significant (*p* < 0.05) results under application of heavy metal Cd^+2^ (500 μM). Considering root soluble phenolics content, it has been observed that the best and effective treatment was SA spray under control at Tbt; however, the less effective treatment was recorded H_2_O spray under Cd^+2^ stress at the same altitude (Figures 6C,D).

The order of improvement in root phenolics contents in Qta was SA spray control > H_2_O_2_ priming control > H_2_O spray control > H_2_O_2_ P + FS control > SA priming Cd^+2^ > SA P + FS Cd^+2^ > H_2_O spray Cd^+2^ > H_2_O_2_ spray Cd^+2^ > H_2_O P + FS Cd^+2^ > SA spray Cd^+2^ > Control Cd^+2^ > Control of Control > SA P + FS control > H_2_O priming Cd^+2^ > H_2_O_2_ spray control > H_2_O_2_ P + FS Cd^+2^ > H_2_O priming control > H_2_O P + FS control > H_2_O_2_ priming Cd^+2^ > SA priming control.

While the order of changes at Tbt was SA spray control > H_2_O_2_ priming Cd^+2^ > H_2_O spray control > SA priming Cd^+2^ > Control of Control > H_2_O_2_ P + FS control > SA spray Cd^+2^ > Control Cd^+2^ > H_2_O P + FS Cd^+2^ > H_2_O_2_ spray control > H_2_O_2_ P + FS Cd^+2^ > H_2_O_2_ spray Cd^+2^ > SA P + FS control > H_2_O_2_ priming control > SA P + FS Cd^+2^ > SA priming control > H_2_O P + FS control > H_2_O priming control > H_2_O priming Cd^+2^ > H_2_O spray Cd^+2^.

On the other hand, Soluble phenolics in shoots reported maximum content in H_2_O_2_ P + FS under heavy metal (Cd^+2^) toxicity at Tbt, while the minimum phenolics content was reported in H_2_O priming under Cd^+2^ toxicity at Qta (Figure 6C).

The order for shoot phenolics content at Qta was observed as follows: H_2_O_2_ spray control > SA priming Cd^+2^ > H_2_O P + FS Cd^+2^ > SA P + FS control > H_2_O_2_ P + FS control > SA spray Cd^+2^ > SA spray control > Control of Control > H_2_O spray control > H_2_O_2_ spray Cd^+2^ > SA P + FS Cd^+2^ > H_2_O P + FS control > H_2_O spray Cd^+2^ > H_2_O priming control > SA priming control > Control Cd^+2^ > H_2_O_2_ priming control > H_2_O_2_ priming Cd^+2^ > H_2_O_2_ P + FS Cd^+2^ > H_2_O priming Cd^+2^.

However, at Tbt, the order of improvement was recorded as H_2_O P + FS Cd^+2^ > H_2_O_2_ spray Cd^+2^ > SA spray Cd^+2^ > SA P + FS Cd^+2^ > Control of Control > SA priming control > SA spray control > H_2_O spray control > SA spray Cd^+2^ > SA P + FS control > H_2_O_2_ spray control > H_2_O spray Cd^+2^ > H_2_O_2_ priming Cd^+2^ > H_2_O_2_ P + FS control > H_2_O priming Cd^+2^ > SA priming Cd^+2^ > H_2_O_2_ priming control > H_2_O priming control > H_2_O priming control > H_2_O P + FS Cd^+2^ > Control Cd^+2^.

Considering the overall observed results of soluble phenolics contents in both roots and shoots, it has been observed that the maximum phenolic content was observed in shoots as compared to the roots. Data pertaining to this attribute revealed that foliar spray and priming with plant signaling molecules (SA and H_2_O_2_) played a significant role in reducing the Cd^+2^ toxicity and enhanced the phenolics content in both roots and shoots. Supplementation with SA and H_2_O_2_ had a stronger effect on the attribute of milk thistle under Cd^+2^ stress as compared to control plants (Figures 6C,D).

#### Tannins Content

Results obtained for Tannins content in both roots and shoots showed statistically significant results (p < 0.05) in milk thistle grown at two different altitudinal fields (i.e., Qta and Tbt) under Cd^+2^ toxicity (500 μM). Data revealed that, in the roots, the highest content of tannins was recorded in SA spray under Cd^+2^ stress at Tbt, while the lowest tannins content was observed in control treatment of Cd^+2^ at the same altitude (Tbt) (Figures 6E,F).

However, the trend for root tannins contents in Qta was observed as H_2_O_2_ P + FS Cd^+2^ > H_2_O_2_ priming Cd^+2^ > H_2_O_2_ priming Cd^+2^ > SA spray Cd^+2^ > H_2_O priming Cd^+2^ > H_2_O spray Cd^+2^ > H_2_O_2_ spray Cd^+2^ > H_2_O P + FS Cd^+2^ > SA priming Cd^+2^ > SA P + FS Cd^+2^ > H_2_O_2_ spray control > Control of Control > SA spray control > H_2_O P + FS control > H_2_O spray control > Control Cd^+2^ > H_2_O_2_ P + FS control > SA priming control > SA P + FS control > H_2_O priming control > H_2_O_2_ priming control.

Considering Tannin content in roots of milk thistle grown at Tbt, following order of improvement was observed: SA spray Cd^+2^ > H_2_O_2_ P + FS Cd^+2^ > H_2_O P + FS Cd^+2^ > SA P + FS Cd^+2^ > H_2_O_2_ spray Cd^+2^ > H_2_O spray Cd^+2^ > H_2_O P + FS control > H_2_O_2_ P + FS control > SA priming Cd^+2^ > SA spray control > H_2_O_2_ spray control > SA P + FS control > H_2_O priming Cd^+2^ > SA priming control > H_2_O_2_ priming Cd^+2^ > Control of Control > H_2_O spray control > H_2_O priming control > H_2_O_2_ priming control > Control Cd^+2^.

Moreover, data recorded for shoot tannins in milk thistle also showed significant variations (*p* < 0.05) under Cd^+2^ toxicity at both altitudes (Qta and Tbt). Furthermore, the obtained results revealed that the maximum tannin contents in shoot were found in H_2_O_2_ priming under Cd^+2^ toxicity at Qta, while the minimum shoot tannins were observed in control at the same altitude (Figure 6E).

The order of improvement in shoot tannins at Qta was observed as H_2_O_2_ priming Cd^+2^ > SA priming Cd^+2^ > H_2_O spray Cd^+2^ > H_2_O P + FS Cd^+2^ > H_2_O_2_ P + FS Cd^+2^ > SA P + FS Cd^+2^ > H_2_O priming Cd^+2^ > SA spray Cd^+2^ > Control Cd^+2^ > H_2_O_2_ spray Cd^+2^ > H_2_O_2_ priming control > H_2_O_2_ spray control > H_2_O_2_ P + FS control > SA spray control > SA priming control > H_2_O priming control > H_2_O spray control > SA P + FS control > H_2_O P + FS control > Control of control.

While the trend for Tbt was recorded as follows: H_2_O P + FS Cd^+2^ > H_2_O_2_ P + FS Cd^+2^ > SA P + FS Cd^+2^ > H_2_O spray Cd^+2^ > H_2_O_2_ spray Cd^+2^ > SA spray Cd^+2^ > SA priming Cd^+2^ > H_2_O_2_ priming control > H_2_O_2_ priming Cd^+2^ > Control Cd^+2^ > SA spray control > H_2_O_2_ P + FS control > H_2_O priming Cd^+2^ > SA P + FS control > SA priming control > H_2_O P + FS control > H_2_O_2_ spray control > H_2_O priming control > H_2_O spray control > Control of Control. In a nutshell, the data statistically revealed significant results by applying plant-signaling molecules in alleviating Cd^+2^ toxicity in milk thistle grown under two varying altitudes, i.e., Qta and Tbt. Compared to control field plants at both altitudes, the Cd^+2^ stress plants showed significantly enhanced tannin content in milk thistle, thus indicating the activation of the defensive mechanism by the synthesis of secondary metabolites (Figures 6E,F).

### Correlations With Shoot and Root Dry Weights

Drawing correlations of various morpho-physiological attributes with shoot and root dry weights (Table 2) revealed that shoot length positively significantly correlated with the dry weights even under Cd^+2^ stress; however, root length showed a negative non-significant correlation under stress. Considering milk thistle leaf area’s correlation with shoot dry weights revealed that, in Qta, under unstressed conditions, the leaf area positively correlated, while, in Tbt, under cadmium stress, the leaf area positively correlated with the SDW. Photosynthetic pigments, i.e., chlorophyll *a*, *b*, and carotenoids showed non-significant correlations except for Chl *a* at Qta in control milk thistle plants that showed significant positive correlations. Secondary metabolites showed non-significant correlations with the shoot and root dry weights under both control and Cd^+2^ stress except for shoot soluble phenolics and root tannin showing significant negative correlation under control conditions and Cd^+2^ stressed plants, respectively, of the Tbt area.

**TABLE 2:** Correlation of changes in various morpho-physiological attributes of milk thistle with shoot and root dry weights grown at Quetta and Turbat under cadmium stress (*n* = 10).

Parameter	Stress	Area	
		Quetta	Turbat
Root length	Control	0.681*	0.157^ns^
	Cd^+2^	–0.380^ns^	–0.050^ns^
Shoot length	Control	0.943**	0.774**
	Cd^+2^	–0.254^ns^	0.760*
Leaf area	Control	0.957**	0.588^ns^
	Cd^+2^	0.351^ns^	0.881**
Root fresh weight	Control	0.824**	0.921**
	Cd^+2^	–0.09^ns^	0.781**
Shoot fresh weight	Control	0.909**	0.911**
	Cd^+2^	0.076^ns^	0.993**
Chlorophyll *a*	Control	0.717*	–0.411^ns^
	Cd^+2^	0.303^ns^	–0.156^ns^
Shoot soluble phenolics	Control	0.322s	–0.649*
	Cd^+2^	–0.323^ns^	–0.325^ns^
Root tannins	Control	–0.296^ns^	0.379^ns^
	Cd^+2^	0.374^ns^	–0.750**
Shoot MDA	Control	–0.024^ns^	–0.193^ns^
	Cd^+2^	–0.037^ns^	0.758*

Significant at: *p < 0.05; **p < 0.01, and ^ns^p > 0.05.

## Discussion

The results of this study on milk thistle illustrate the significant role of plant-signaling molecules SA and H_2_O_2_ in alleviating Cd^+2^ toxicity at two varying altitudes of Balochistan (Qta and Tbt), Pakistan. However, the priming and foliar spray of SA and H_2_O_2_ enhanced the germination, morpho-physiological effectiveness at both altitudes, thus thereby ameliorating the toxic effect of Cd^+2^ in milk thistle. Cd^+2^ is widely recognized as a toxic heavy metal in soil by destabilizing the integrity of membrane and status of nutrients; that inhibits biosynthesis of chlorophyll that resulted in the reduction in plant growth and development (Lu et al., 2018; Majumdar et al., 2020). Considering the growth attributes of the present study, it was observed that roots, shoot length, and weights decreased under Cd^+2^ toxicity; however, the supplementation of SA and H_2_O_2_ helps the milk thistle plants to alleviate the toxic impacts in single or combinational treatments at both experimental sites. Khatamipour et al. (2011) reported the various Cd^+2^ concentrations significantly reduce root length in milk thistle. The decline in root length was more pronounced because of the Cd^+2^ treatment as compared to shoot length. The toxicity of Cd^+2^ was previously reported in different plant species and groups (Kaur et al., 2017; Lu et al., 2018; Kaya et al., 2019; Majumdar et al., 2020). The attributes for growth in *Brassica juncea*, such as shoot/root length, dry and fresh weight, along with the leaf area, decreased by the application of Cd^+2^ (Faraz et al., 2020). However, the findings of Migahid et al.’s (2019) research suggested that priming of seeds with H_2_O_2_ increased the leaf area, dry weight of roots and shoots, and root and shoot fresh weight of milk thistle (Gaertn) in comparison with control (Wahid et al., 2007). Furthermore, in the present study, it has been reported that Cd^+2^ toxicity decreased the leaf area at high altitude (Qta), but by the priming and foliar application of plant-signaling molecules (i.e., SA and H_2_O_2_), the morphological parameters (i.e., root/shoot length, the number of leaves, shoot and root fresh weight) increased and also alleviated the toxic effects of heavy metal cadmium (Figures 2A–C, 3A,D).

The application of Cd^+2^ concentration reduces the rate of photosynthesis by targeting various electron transport chain components in carboxylation reactions along with PSII (Shah et al., 2020). Study, conducted by Guo et al. (2019) also reported a decrease in contents of chlorophyll and fluorescence under Cd^ + 2^ toxicity. In this study, milk thistle plants treated with priming and foliar spray of SA and H_2_O_2_ have a propounding effect on alleviating the toxicity of Cd^+2^ on the photosynthetic pigments (Figures 4A–C). The carotenoid contents in plants were reduced because of high cadmium Cd^+2^ (Cai et al., 2011), while our findings showed non-significant (*p* > 0.05) results under both control and Cd^+2^ conditions. Carotenoid contents are enhanced due to plant-signaling molecules (SA and H_2_O_2_) at high- (Qta) and low-altitude Tbt (Figure 4C). Carotenoids are the potent antioxidants that serve as reactive oxygen species (ROS) against disruption of photo-oxidative in photosystems (Zahra et al., 2021d), thus suggesting the putative role of SA and H_2_O_2_ in improving plant defense mechanisms.

MDA is considered the main product for peroxidation of the lipid membrane. Contents of MDA reflect the damage to structures of cell membranes (Parida and Jha, 2010). While, under Cd^+2^ toxicity, high-level MDA accumulation is reported to enhance O_2_^–^ and H_2_O_2_ (Wang et al., 2013a; Xu et al., 2014). Furthermore, Krantev et al. (2008) reported that seeds of maize were presoaked with 500 mM SA before applying the Cd^+2^ has a protective effect by diminishing MDA accumulation, which is involved in the protection against oxidative damage. Wang et al. (2013a,b) and Bai et al. (2015) also reported pretreatment of SA under cadmium stress-reduced MDA contents. In the present study, it has been reported that MDA content enhanced in roots of milk thistle under Cd^+2^ stress (Figure 5D). Popova et al. (2008) reported that Cd^+2^ was accumulated in roots of the plants because it is the basic organ that was exposed to heavy metals in soil and translocated to shoots as well. Cd^+2^ stress was reported to cause lipid peroxidation in roots of pea seedlings with increasing levels of H_2_O_2_ (Podazza et al., 2012). The outcome of the report was further shown in the study of El Dakak and Hassan (2020) that H_2_O_2_ was not significantly accumulated (*p* > 0.05); hence, our findings support the same findings that H_2_O_2_ content in shoots showed non-significant results in milk thistle under Cd^+2^ stress (Figure 5A). Song et al. (2014) reported enhancing activities of antioxidative enzymes (i.e., SOD and POD) along with some other enzymatic antioxidants such as CAT and APX decreased by the exposure of Cd^ + 2^ toxicity. Liu et al. (2010) reported that Cd^+2^-generated oxidative damage interacts with antioxidant defense system. While the increasing activity of SOD and POD indicated detoxifying of ROS (Ferreira et al., 2002; Krantev et al., 2008). Furthermore, POD activated by Cd^+2^ at a minimum level is responsible for the removal of H_2_O_2_, which insignificantly (*p* > 0.05) increased.

Plant secondary metabolites play a key role in defense against environmental stresses (Yang et al., 2018). A slow process was observed due to secondary metabolites accumulation during the stress conditions in lemongrass, which reported greater accumulation in soluble phenolics, flavonoids, and anthocyanin, while the accumulation of tannins content was minimum both in the shoots and roots (Shaukat et al., 2020) after exposure to different altitudes. Basically, tannins are of two types (i.e., condensed and hydrolysable). The condensed tannins are physiologically less significant due to being much complex, not soluble, but hydrolysable tannins played a significant role in growth attributes along with development under adverse conditions (Tiku, 2020). Such compounds, apart from tannin, are mainly found in the soluble phase, which increases in the concentration of secondary metabolites under adverse environmental conditions that are known (Tiku, 2020). In the present study, it has been observed that accumulation of tannin content in roots and shoots of milk thistle at both altitudes (i.e., Qta and Tbt) enhanced under Cd^+2^ stress. However, at Tbt, the tannins contents in roots were comparatively less than in Qta milk thistle plants, thus indicating environmental regime variation impact (Figures 6E,F).

Phenolics are donors of electrons that could mitigate the oxidative stress effect, which is an ultimate substrate for antioxidant enzymes, i.e., peroxidases (Posmyk et al., 2009). Migahid et al. (2019) reported that the total phenolic contents in milk thistle did not change under H_2_O_2_. However, our findings in the present study suggest that phenolics contents in the roots decrease significantly in control and Cd^+2^ conditions at both altitudes (i.e., Qta and Tbt), while phenolics contents in shoots significantly (*p* < 0.05) increased to a high level as compared to root phenolics, while, under Cd^+2^ stress, the respective treatments applied with a concentration of SA and H_2_O_2_ showed significant results (Figures 6C,D). Chaman et al. (2003) reported that SA increases the phenylalanine ammonia-lyase activity that is an essential enzyme being involved in phenolics compounds biosynthesis in the early stages. Zahra et al. (2021d) reported that, in milk thistle, it was perused that the alkaloid and anthocyanin contents enhanced under stress in comparison with control. It was also reported from the study of Khan et al. (2019) that foliar application of SA increased anthocyanin contents in seedlings of rice subjected to abiotic stress. The same tendency was observed in the present study, that anthocyanin contents in shoot significantly (*p* < 0.05) increased while, in roots, a non-significant (*p* > 0.05) increased under Cd^+2^ toxicity (Figures 6A,B). However, under control conditions, the contents of anthocyanin remain suppressed at both the altitudinal fields Qta and Tbt (Figures 6A,B), thus providing the evidence that the secondary metabolite increases and work as defense system under stressful environments.

## Conclusion

The toxicity of heavy metal (Cd^+2^) hampered the morphological and physiological attributes of milk thistle grown at two different altitudes. Data revealed that, under Cd^+2^ toxicity, morpho-physiological attributes were hampered. However, priming and foliar treatment of plant-signaling molecules SA and H_2_O_2_ showed a positive impact on alleviating the toxic effect of Cd^+2^. The secondary metabolites showed improved tolerance to Cd^+2^ concentration supported the growth and development of milk thistle at both altitudes by modulating Cd^+2^-induced changes, especially with the application of SA and H_2_O_2_. Milk thistle being a weed plant grown in areas, which are mostly heavy metal affected due to industrially released chemicals, mining, and cadmium present in soil; it would be very beneficial to grow these plants, which can tolerate heavy metal stress. The present research is thus helpful to conduct comparative studies of medicinal plants grown under varying altitudes and under cadmium/heavy metal stress. The study motivates to unveil the genes involved in ameliorating the heavy metal toxicity in the same plant across the altitudinal gradient as no such literature has yet been reported.

## Data Availability Statement

The original contributions presented in the study are included in the article/supplementary material, further inquiries can be directed to the corresponding authors.

## Author Contributions

MN and KS conceived and designed the research. MN, KS, and AS performed the experiment and wrote the initial draft of the manuscript. AR, NZ, and MBH assisted with the analysis, revised subsequent versions of the manuscript, and provided the technical guidance. AR, KS, QA, HMA, and MHS proofread and edited the final version. All authors have read and agreed to the published version of the manuscript.

## Conflict of Interest

The authors declare that the research was conducted in the absence of any commercial or financial relationships that could be construed as a potential conflict of interest.

## Publisher’s Note

All claims expressed in this article are solely those of the authors and do not necessarily represent those of their affiliated organizations, or those of the publisher, the editors and the reviewers. Any product that may be evaluated in this article, or claim that may be made by its manufacturer, is not guaranteed or endorsed by the publisher.
